# The Act on Integrated Support for Community Care Including Medical and Nursing Services: Implications for the Role of Tertiary Hospitals in the Republic of Korea

**DOI:** 10.3390/healthcare13101156

**Published:** 2025-05-15

**Authors:** Byeungtae Park, Pyeong-Man Kim, Chul-Min Kim, Chang-Jin Choi, Hyun-Young Shin

**Affiliations:** 1 Graduate School of Public Health and Healthcare Management, The Catholic University of Korea, Seoul 06591, Republic of Korea; 2 Department of Humanities and Social Medicine, College of Medicine, The Catholic University of Korea, Seoul 06591, Republic of Korea; 3 Department of Family Medicine, Seoul St. Mary’s Hospital, College of Medicine, The Catholic University of Korea, Seoul 06591, Republic of Korea

**Keywords:** tertiary hospital, community care, family medicine, digital healthcare

## Abstract

**Background/Objectives:** The Republic of Korea is undergoing a significant demographic shift toward a population with a high proportion of older adults. In response, the *Act on Integrated Support for Community Care* was enacted. This study explores the role of tertiary hospitals in integrated care, aiming to enhance healthcare systems that support older individuals by facilitating the transition from hospital- to community-based care. **Methods:** Seoul St. Mary’s Hospital, operating under the Catholic Foundation, provides care grounded in healing and spirituality. As part of its mission, a multidisciplinary task force (TF) was formed to examine the hospital’s role in integrated care for an aging society. The TF, composed of eight experts from various departments, engaged in open discussions from September 2024 to January 2025. **Results:** The Integrated Care Act, which seeks to integrate medical care and caregiving within communities, requires the development of a digital system, the establishment of a governance framework for multidisciplinary collaboration, and the creation of institutions for training professionals in integrated care. Tertiary hospitals must develop department-specific models for transitional care and establish policy research institutes focused on holistic, patient-centered care. Family medicine departments can play a central role in coordinating between tertiary hospitals and local communities. **Conclusions:** This study highlights the importance of collaboration between medical, caregiving, and social welfare professionals as key enablers of “aging in place”. The findings underscore the evolving role of tertiary hospitals and contribute to fostering a more sustainable healthcare model for Korea’s aging population.

## 1. Introduction

In 2025, the Republic of Korea entered a super-aged society, with individuals aged 65 and older accounting for 20.3% of the population—meaning one in five people will be elderly. According to the 2024 Statistics on the Older Adults in Korea, 2.138 million elderly households are living alone, and 18.7% of them have no one to support their daily lives [1]. With the growing elderly population, concerns are rising over the accelerated deterioration of the National Health Insurance System’s financial stability, particularly as medical expenses increase significantly in the last three months of life [2].

In response, the National Assembly of Korea passed a law in 2024 to integrate medical and long-term care support within local communities, with on-site implementation scheduled for 2026 (No, 2208590, 26 March 2024) [3]. Various policies and pilot projects related to community care, home medical services, and integrated care are currently underway [4]. Efforts are particularly focused on addressing healthcare gaps for elderly and disabled individuals in local communities who struggle to access medical benefits, including the expansion of home visits by local healthcare institutions.

Additionally, recognizing the need for healthcare reform, the government reinforces the previously weak healthcare delivery system and promotes policy changes that allow tertiary hospitals to focus on critical patient care [5]. In this context, the role of family medicine in tertiary hospitals gains attention, particularly as home medical care becomes increasingly essential in an aging society. Family medicine is expected to serve as a crucial bridge in transitioning patients from tertiary hospitals back to local communities.

Home medical care, when combined with digital healthcare, serves as a cornerstone for implementing the concept of “aging in place” within communities. It has the potential to become a testbed for future healthcare policies, facilitating a shift toward patient-centered care, where individuals receive medical benefits in the comfort of their own homes [6,7].

As societies confront the challenges of an aging population, the development of long-term medical care systems and the transition toward integrated care occur concurrently across various countries. In England, the evolution of Integrated Care Systems (ICS) began with the vanguard projects in 2014 and was formally established through *the Health and Care Act, 2022*. This framework seeks to enhance coordination between hospitals, general practitioners, and social care services by integrating National Health Service (NHS) organizations with local government bodies [8]. Japan’s Integrated Community Care System, introduced in 2017, prioritizes community-based services that facilitate aging in place. The system integrates health and social care through neighborhood-based networks, promoting collaboration among service providers to address the diverse needs of the elderly population [9].

This study aims to establish a foundation for a comprehensive care system in an aging society, benefiting both patients and their caregivers. It seeks to define the functions and roles of tertiary hospitals in the era of integrated care, based on a project led by a task force within a single tertiary hospital. Furthermore, it highlights the need to initiate discussions on managing pilot projects for a future integrated network model that supports patients’ transition back into the community after hospital discharge. This will help expand the positive impact of advanced healthcare models that integrate medical care, health management, caregiving, and spiritual support.

## 2. Materials and Methods

Seoul St. Mary’s Hospital, one of the five largest hospitals in Korea, operates under the Catholic Foundation, in the ideology of healing and spirituality. As part of its mission as both a university and tertiary hospital, a multidisciplinary task force (TF) was formed to explore the hospital’s role in integrated care with community for an aging society.

The TF consisted of eight experts specializing in home care, including representatives from the Department of Family Medicine, Department of Nursing, Family Nursing Center, Spiritual Medicine, and the Graduate School of Health and Medical Management. The team was composed of a physician (professor of Family Medicine), a nurse (professor at a Nursing College), a healthcare management expert (a former government official and a professor specializing in Service Design), a home care team working in secondary and tertiary hospitals, a professor of the Humanities and Social Sciences, and priests and nuns for implementing Spiritual Medicine. The TF members conducted a descriptive analysis of the history, preconditions, and roles of relevant medical institutions in the integrated care method by reviewing the literature and related documents published in the past 10 years. The meetings were held once a month for a total of three times, conducted in a roundtable format with discussions for consensus-building with experts, lasting two hours. In the fourth meeting, a draft for organizing opinions was prepared, and feedback was collected to establish the final proposal. Discussions were conducted using an open, free discussion format to examine the hospital’s potential contributions to integrated care.

The TF convened from September 2024 to January 2025, holding both offline and online meetings. The following topics were discussed:

(1). Analysis of *the Act on Integrated Support for Community Care Including Medical Cure and Nursing Care* (hereinafter referred to as the *Integrated Care Act*).

(2) Evaluation of the government’s home care policy and projections for the future direction of healthcare.

(3) Establishment of a home care center, including defining its roles and functions and developing a collaborative system with existing home care centers within the hospital.

(4) The role of integrated care in tertiary hospitals, focusing on their connection to primary and secondary care within the community.

## 3. Results

### 3.1. Analysis of the Integrated Care Act

#### 3.1.1. Proposition and Legislative Progress

As Korea transitions into a super-aged society, the social demand for integrated medical and long-term care has steadily increased. This shift in policy aims to move away from the traditional practice of placing elderly individuals in nursing hospitals or care facilities at the end of life, instead promoting a model that integrates medical care and caregiving within communities.

During the 21st National Assembly (2020–2024), seven legislators from both ruling and opposition parties proposed bills related to integrated care. Following a public hearing on the enactment of Integrated Care Act, the bill was passed through the Subcommittee on Bills of the Health and Welfare Committee, the Legislative Judicial Committee, and the plenary session. The law was officially promulgated on 26 March 2024, and its full implementation is scheduled for 27 March 2026 [3,10].

#### 3.1.2. Prerequisites for the Introduction and Implementation of the Integrated Care Act

The Integrated Care Act consists of seven chapters and 30 articles, outlining [11]:

(1) The establishment of a basic plan for integrated support;

(2) Procedures for policy implementation and support;

(3) The development of an integrated care infrastructure.

The successful implementation of the Act requires significant preparation in terms of time, effort, and budget. Key prerequisites include:

(1) Development of an integrated digital system for managing health and welfare data of individuals receiving integrated care;

(2) Establishment of a governance framework for multidisciplinary collaboration among medical, caregiving, and welfare professionals;

(3) Creation of central and local government departments responsible for overseeing integrated care;

(4) Designation and development of institutions specializing in training professional personnel for integrated care [7,11].

The key healthcare aspects of the law are outlined in Table 1.

### 3.2. The Role of Tertiary Medical Institutions in Integrated Care Based on the Integrated Care Act

#### 3.2.1. Strengthening Transitional Care

Implementation of transitional care is necessary to enhance intensive care management and reduce hospital stays, thereby improving the fundamental function of tertiary hospitals [12,13,14]. With the increasing number of geriatric patients, including those with mobility impairments and age-related conditions, transitional care must be reinforced to facilitate post-discharge patient recovery in preparation for a super-aged society [15,16]. A department-specific transitional care model should be developed, addressing diseases in neurology, rehabilitation medicine, psychiatry, orthopedic surgery, hematologic oncology, pediatrics, hospice care and family medicine. Additionally, a protocol should be established to ensure seamless linkage between hospital care and home medical services [17]. Home medical linkage programs should be designed for mobility restricted patients, including those with Parkinson’s disease, amyotrophic lateral sclerosis (ALS), dementia, postoperative conditions, disabilities, elderly individuals living alone, and terminally ill patients, including those with advanced cancer, requiring end-of-life care [18]. A cooperative system must be established to integrate discharge planning with continuous care, ensuring collaboration between home medical services and social workers to create a joint discharge plan [19].

#### 3.2.2. Establishment of a Home Medical Center

Given the growing elderly population and the increasing financial strain on health insurance, government healthcare policies will continue to expand and accelerate initiatives related to community care and integrated care support. A Home Medical Center could be established within tertiary hospitals, with a clear role for the family medicine department to adapt to evolving healthcare policies [20,21]. A system should be developed to facilitate end-of-life dignity through hospice and palliative care services within the community. This requires collaboration between home medical centers and palliative care departments to develop specialized services.

Policy support for issuing death certificates at home is needed to enable elderly patients with non-hospice-qualifying illnesses to pass away in familiar environments rather than in facilities. This initiative should be proactively proposed to the government and integrated with bereavement care services for families [22].

Establishing a Community Cooperation System [23]:

(1) Develop a network linking primary and secondary hospitals to enhance patient referrals.

(2) Strengthen collaboration between hospitals, local health centers, and local governments.

(3) Optimize referrals from tertiary hospitals by reinforcing a coordinated care pathway.

(4) Create a cooperative framework with primary healthcare providers, including scheduled visits (monthly or quarterly) and regular multidisciplinary meetings.

(5) Enhance efficiency through digital healthcare integration, including telemedicine, remote patient monitoring, home visits, and consultations [24].

(6) Monitoring and long-term management of community-based patients can be strengthened through structured home care programs, emphasizing continuous care and patient severity assessment within an integrated system.

#### 3.2.3. Establishing a Policy Research Institute and Integrated Human Resource Training

(1) A policy research institute can serve as a specialized institution that collaborates with the government, local authorities, and community organizations to support home care projects. Its role would include institutional design, evaluation, workforce training, and education.

(2) As a specialized entity under the Integrated Care Act, this institution should oversee:

- Policy development and research initiatives;
- Promotion of integrated care and support for regional plan performance evaluations;
- Identification and classification of patient groups based on disease characteristics;
- Comprehensive assessment of pilot projects to enhance implementation strategies;
- Legal and institutional review for a smoother transition from pilot projects to full-scale implementation [4].

(3) Particularly, religious-affiliated hospitals can offer a unique and differentiated approach in establishing a patient-centered, holistic care system that integrates medical treatment, caregiving, and spiritual support, ensuring a dignified life and end-of-life experience [18].

By leveraging the collaborative influence of healthcare professionals, religious organizations, and caregivers, these institutions can play a pivotal role in developing, implementing, and evaluating integrated care programs that foster open communication, interdisciplinary synergy, and harmonized service delivery.

## 4. Discussion

Korea is undergoing a rapid demographic shift toward an aging society, necessitating the advancement of patient-centered integrated care to ensure a dignified end-of-life experience. In February 2024, the National Assembly passed the Act on Integrated Support for Community Care Including Medical Cure and Nursing Care, and, starting in 2026, both the national and local governments will make comprehensive efforts to implement its provisions [3]. However, from the patient’s perspective, those experiencing immobility are often left with no choice but to enter nursing hospitals or long-term care facilities. This situation arises due to the fragmented nature of national health insurance and long-term care insurance, leading to inefficiencies in service delivery. Additionally, overlapping pilot projects and a lack of continuity in home care services further highlight the need for a comprehensive institutional reform and fundamental improvements in the system [25].

In response to the global aging population, many countries have been continuously working to integrate fragmented healthcare and long-term care services. As nations across Asia, including Japan, South Korea, China, Thailand, and Indonesia, continue to reform their healthcare and long-term care systems, the direction of transformation varies [26]. Japan is shifting toward primary healthcare, with significant reforms underway, while China focuses on facility-based care integration, and Thailand prioritizes home-based care [9,26]. Japan’s Integrated Community Care System, introduced in 2017, emphasizes community-based services to support aging in place, utilizing neighborhood care networks to integrate health and social care. China’s long-term care system is characterized by a rapid expansion of residential care facilities, although home- and community-based services have developed more slowly [27]. Public financing remains limited, primarily supporting welfare recipients and subsidizing residential care, with pilot programs exploring social insurance models. Thailand has developed a home care model that prioritizes community-based services, relying on healthcare professionals and volunteers to support caregivers and manage chronic diseases among the elderly.

As Korea undergoes medical reform, the role of tertiary hospitals in integrated care must be re-evaluated. The current healthcare framework focuses on strengthening tertiary hospitals for acute and severe cases while redirecting referrals to lower-level care facilities. If tertiary hospitals can establish a cycle of hospitalization → rehabilitation → home care → re-hospitalization, they can optimize their role in intensive care while enhancing continuity of treatment [19]. Furthermore, if patients admitted in acute phases receive structured transitional care plans before discharge, their reintegration into society and independent living can be accelerated, reducing hospital stays. Since patients’ disease characteristics and socio-economic conditions vary, an integrated care program must be tailored to individual needs [28,29].

A crucial component of this system is the continuous linkage between home care services and hospital-based care. By integrating visiting medical services and home nursing, unnecessary hospital visits can be minimized, and community-based referrals to primary and secondary care facilities can be strengthened [12,13,14]. To achieve this, tertiary hospitals should establish dedicated home care centers—with the family medicine department as the central coordinating unit—to support an integrated community care model. These centers would not only enhance primary care-driven home care services but also contribute to medical system efficiency, digital healthcare integration, and policy leadership in pilot programs and research [20,21,24].

As tertiary university hospitals move beyond traditional treatment roles, they must engage in healthcare policy discourse in the super-aged era. Several critical questions arise, namely:

(1) How will patient-centered and customized support be implemented in future healthcare models?

(2) Can critically ill patients age in place at home rather than in institutional care?

(3) How can Korea address the persistent shortage of nursing and caregiving services, and prevent caregiver burden and tragic incidents such as caregiving-related homicides?

(4) How much financial and policy support can the government and institutions provide to ensure that home care for elderly patients does not become an unsustainable burden on families?

(5) Can non-face-to-face treatment, digital healthcare advancements, and remote patient monitoring be integrated into existing national health insurance reimbursement models to ensure sustainable, high-quality care?

The concept of “Aging in Place” presents both opportunities and challenges. Korea’s healthcare system must evolve into a nationwide, integrated medical delivery ecosystem, linking medical, nursing, and caregiving services. However, the financial strain on medical institutions and the ongoing healthcare crisis raise concerns about the sustainability of essential services such as emergencies and critical care [30]. Patients expect systemic reforms that will ensure trust in healthcare institutions and allow for a viable long-term solution within the constraints of the national health insurance system. To meet these challenges, tertiary hospitals must establish sustainable models that bridge the gap between transitional care and home care, ensuring a dignified healthcare experience for patients.

As a Catholic Foundation Hospital, Seoul St. Mary’s Hospital is uniquely positioned to integrate medical, spiritual, and holistic care for aging patients. This approach can expand beyond Catholic communities to the broader Korean population, reinforcing the integration of medical care, nursing, and community-based services. The establishment of home medical centers within the Catholic Medical Center could further enhance community-based healthcare networks, enabling collaboration between medical personnel, local parishes, and community care providers.

This study has several limitations: The short study period and the homogeneity of participants, all of whom were experts from the same tertiary hospital, may limit the generalizability of findings. The study was conceptual and exploratory, focusing on brainstorming discussions rather than the actual planning and implementation of an integrated care program. Given the subjective and experience-based nature of expert discussions, the possibility of selective bias cannot be ruled out.

However, the issue of population aging is a global phenomenon, and the restructuring of the healthcare system in Korea, in response to the aging era, is expected to provide valuable empirical lessons as a good precedent. The demand for changes in the healthcare system to address the health of local residents is a common challenge for all countries, and it is believed that sharing coping know-how and fostering collaboration within the academic community will become increasingly important.

## 5. Conclusions

Korea’s aging healthcare system is at a critical turning point, requiring flexible approaches and systemic changes to implement advanced medical care. It is essential to foster ongoing policy discussions, strengthen professional competencies, and implement patient-centered integrated care programs. Tertiary hospitals must take a proactive role in preparing for the future of healthcare by embracing integrated care and home medical services. Moving forward, collaboration between policymakers, academics, and healthcare leaders will be crucial in shaping a sustainable and efficient medical delivery system, ensuring effective integration between primary, secondary, and home care services.

## Figures and Tables

**Table 1:** Key healthcare provisions of the Act on Integrated Support for Community Care Including Healthcare and Long-Term Care.

Article 15 (Healthcare)	① The national and local governments must strive to expand healthcare services that align with the needs of integrated support recipients and enhance connections with other services. (1) Medical services provided by doctors, dentists, and oriental medicine doctors (2) Nursing services provided by nurses (3) Rehabilitation services (4) Medical services in long-term care hospitals and other facilities (5) Hospice care programs (6) Home-based oral health management (7) Medication guidance (8) Other services specified by the Ministry of Health and Welfare
Article 16 (Health Management and Prevention)	① The national and local governments must work to prevent or mitigate frailty, geriatric diseases, chronic illnesses, disabilities, and mental disorders among integrated support recipients while supporting daily living and health management. ② Local governments must establish an integrated support system that enables interdisciplinary cooperation between healthcare, nursing, and welfare services for effective health management and prevention.
Article 20 (Integrated Support Council)	① The governor of a province (Si/Do) or the mayor, county chief, or district head (Si/Gun/Gu) must establish an Integrated Support Council within their jurisdiction to facilitate the effective implementation of integrated support and strengthen cooperation with related institutions. ② The Integrated Support Council shall deliberate and provide advice on the following matters: (1) Development and evaluation of regional plans (2) Implementation of integrated support policies (3) Cooperation and coordination with integrated support-related institutions (4) Other matters deemed necessary by the chairperson
Article 24 (Training of Specialized Personnel)	① The state and local governments must develop policies to train, secure, and enhance the quality of specialized personnel required for integrated support ② The state may designate research institutes, universities, or other necessary institutions as specialized training and retraining centers for integrated support professionals
Article 25 (Designation of Specialized Institutions)	① To efficiently identify integrated support recipients, collect, investigate, and analyze related data, and promote integrated support policies, the Minister of Health and Welfare may designate specialized institutions to perform the following tasks: (1) Assist in the development, promotion, and evaluation of national and local integrated support policies (2) Analyze the characteristics and classifications of integrated support recipients (3) Support recipient identification and investigation, and develop assessment criteria (4) Conduct comprehensive evaluations (5) Other necessary tasks specified by the Ministry of Health and Welfare
Article 26 (Pilot Programs)	① If full-scale implementation of the integrated support system is expected to be challenging or requires prior validation of operational methods, the Minister of Health and Welfare may conduct pilot projects in advance

## Data Availability

No new data were created or analyzed in this study. Data sharing is not applicable to this article.

## Abbreviations

The following abbreviations are used in this manuscript:

TF	task force
Integrated Care Act	Act on Integrated Support for Community Care Including Medical Cure and Nursing Care
